# SPEN induces miR-4652-3p to target HIPK2 in nasopharyngeal carcinoma

**DOI:** 10.1038/s41419-020-2699-2

**Published:** 2020-07-02

**Authors:** Yang Li, Yumin Lv, Chao Cheng, Yan Huang, Liu Yang, Jingjing He, Xingyu Tao, Yingying Hu, Yuting Ma, Yun Su, Liyang Wu, Guifang Yu, Qingping Jiang, Shu Liu, Xiong Liu, Zhen Liu

**Affiliations:** 1 Affiliated Cancer Hospital & Institute of Guangzhou Medical University, Guangzhou Municipal and Guangdong Provincial Key Laboratory of Protein Modification and Degradation, State Key Laboratory of Respiratory Disease, School of Basic Medical Sciences, Guangzhou Medical University, Guangzhou, 510095 Guangdong China; 2 Department of Pediatric Otorhinolaryngology, Shenzhen Key Laboratory of Viral Oncology, The Clinical Innovation & Research Centre, Shenzhen Hospital, Southern Medical University, Shenzhen, Guangdong China; 3 Department of Pathology, The Sixth Affiliated Hospital of Sun Yat-sen University, Guangzhou, Guangdong China; 4 Department of Oncology, The Fifth Affiliated Hospital of Guangzhou Medical University, Guangzhou, Guangdong China; 5 Department of Pathology, Third Affiliated Hospital of Guangzhou Medical University, Guangzhou, Guangdong China; 6 Department of Breast Surgery, Guiyang Maternal and Child Healthcare Hospital, Guiyang, 550003 Guizhou China; 7 E.N.T. Department of Nanfang Hospital, Southern Medical University, Guangzhou, Guangdong China

**Keywords:** Cancer, Cell invasion

## Abstract

SPEN family transcriptional repressor (SPEN), also known as the SMART/HDAC1-associated repressor protein (SHARP), has been reported to modulate the malignant phenotypes of breast cancer, colon cancer, and ovarian cancer. However, its role and the detail molecular basis in nasopharyngeal carcinoma (NPC) remain elusive. In this study, the SPEN mRNA and protein expression was found to be increased in NPC cells and tissues compared with nonmalignant nasopharyngeal epithelial cells and tissues. Elevated SPEN protein expression was found to promote the pathogenesis of NPC and lead to poor prognosis. Knockdown of SPEN expression resulted in inactivation ofPI3K/AKT and c-JUN signaling, thereby suppressing NPC migration and invasion. In addition, miR-4652-3p was found to be a downstream inducer of SPEN by targeting the homeodomain interacting protein kinase 2 (HIPK2) gene, a potential tumor suppressor that reduces the activation of epithelial–mesenchymal transition (EMT) signaling, thereby reducing its expression and leading to increased NPC migration, invasion, and metastasis. In addition, SPEN was found to induce miR-4652-3p expression by activating PI3K/AKT/c-JUN signaling to target HIPK2. Our data provided a new molecular mechanism for SPEN as a metastasis promoter through activation of PI3K/AKT signaling, thereby stimulating the c-JUN/miR-4652-3p axis to target HIPK2 in NPC.

## Introduction

Nasopharyngeal carcinoma (NPC) is highly prevalent in Southern China with a much higher incidence than elsewhere^1^. It is characterized by high invasion and early metastasis. Patients with NPC are often diagnosed at advanced stage of the disease^2^. Although regional control has been greatly improved by the advances in radiotherapy and chemotherapy, metastasis remains the major cause of treatment failure^3^. Accordingly, understanding the molecular mechanisms by of invasion and/or metastasis of NPC is critical for the identification of novel therapeutic targets and formulation of better treatment strategies.

In previous studies, several genes have been reported to be involved in NPC metastasis^4–6^. SPEN family transcriptional repressor (SPEN), also known as SMART/HDAC1-associatedrepressor (SHARP), is a large nuclear protein that plays an important role in transcriptional regulation and inactivation of chromosome X^7^. Legare et al. reported that the inactivation of *SPEN* may contribute to breast tumor progression and thus suggested SPEN as a tumor suppressor in ERα-positive breast cancers^8^. In contrast, Feng et al. found that SPEN (SHARP) gene acts as a candidate oncogene, promoting the pathogenesis of human hematopoietic malignancies, breast and colon cancer^9^. Furthermore, Liu et al. demonstrated that SPOCD1(SPEN) may act as a carcinogenesis factor by activating the PI3K/AKT pathway to restrained cell apoptosis in Ovarian cancer (OC)^10^. These studies suggested that SPEN played a significant and complexed role in tumor pathogenesis. However, the molecular alterations and biological functional involvement of SPEN in the pathogenesis of NPC have not been investigated.

MicroRNAs (miRNAs) area class of small (17–23 nucleotides) noncoding RNAs that silence mRNA molecules through a degradation or translational inhibition process. They participate in various biological processes, including tumorigenesis and metastasis^11–13^. Multiple miRNAs have been found to play key roles in regulating the expression of various critical genes during the development of human tumors^4,14,15^. Several of them were identified as regulators of the progression of NPC, such as miR-374a, miR-184, and miR-3188^6,16,17^. However, the regulation of miRNAs involving SPEN has not been reported to date.

This study reports a newly discovered miRNA, namely miR-4652-3p, as an oncogenic regulator miRNA, which was found to be upregulated by the potential oncogene SPEN through the activation of PI3K/AKT/c-JUN signaling. In addition, miR-4652-3p was found to directly target *HIPK2* to participate in the SPEN-mediated promotion of NPC migration, invasion, and metastasis.

## Results

### SPEN expression and clinicopathological characteristics in NPC

**Fig. 1:**
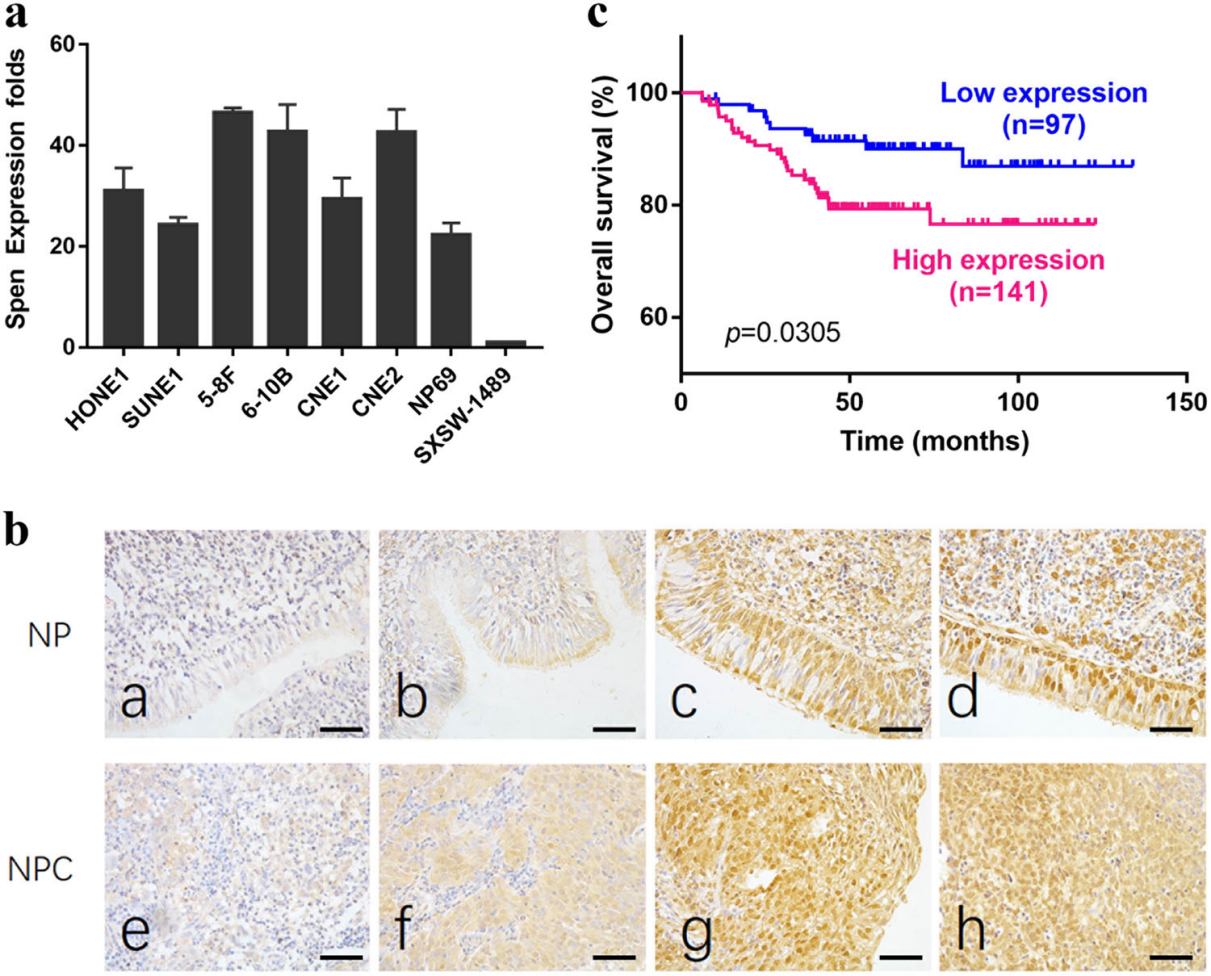
Elevated expression of SPEN promoted the poor prognosis of NPC patients. **a** mRNA levels of *SPEN* analyzed by qRT-PCR assays in six human NPC cell lines (HONE1, SUNE1, 5–8F, 6-10B, CNE1, CNE2) and immortalized normal nasopharyngeal epithelial cell lines NP69 and SXSW-1489. **b** Representative IHC images of SPEN expression in NP and NPC tissues. a, b: weak expression of SPEN in NP samples; c, d: strong and positive expression of SPEN in NP samples; e, f: weak staining of SPEN in NPC specimens; g, h: strong and positive staining of SPEN in NPC specimens. (original magnification ×400). **c** Kaplan–Meier survival curve for overall survival in NPC patients based on SPEN expression level (*P* = 0.0305, log-rank test).

To determine the role of *SPEN* in NPC development, its expression level was analyzed in various NPC cell lines (HONE1, SUNE1, 5-8F, 6-10B, CNE1, and CNE2) and immortalized nasopharyngeal epithelial (NP) cell lines (NP69 and SXSW-1489) by quantitative real-time polymerase chain reaction (qRT-PCR) analysis. The endogenous mRNA level of *SPEN* in all six NPC cell lines was significantly upregulated compared with that in SXSW-1489 nonmalignant immortalized NP cells, although the difference between NPC cells and NP69 nonmalignant NP cells (Fig. 1a) was not significant. As for protein level, a large cohort of 238 NPC tissues and 54 nonmalignant NP tissues were examined by immunohistochemistry (IHC) analysis. SPEN expression displayed nuclear and cytoplasmic distribution patterns in both NPC and NP cells with different expression levels (Fig. 1b). Statistical analysis confirmed that among the 238 NPC specimens, 97 (40.8%) had low SPEN expression and 141 (59.2%) had high SPEN expression. Instead, among the 54 NP tissues, low SPEN-expressing tissues accounted for 44 (81.5%), and high SPEN-expressing tissues accounted for 10 (18.5%). In addition, NPC tissues showed higher SPEN expression level than NP tissues(*P* < 0.001, Table 1). In addition, the analysis of the relationship between SPEN expression and the clinicopathological characteristics in patients with NPC revealed no statistically significant association between *SPEN* expression level and patient age and gender, although SPEN expression was positively correlated with the N (lymph node metastasis) stage (*P* < 0.001; N0-N1 vs. N2–N3), *T* (tumor size) stage (*P* = 0.021; T1–T2 vs. T3–T4) and clinical stage (*P* < 0.001; I–II vs. III–IV, Table 2). Survival analysis revealed that low-SPEN expressing patients had longer overall survival than high SPEN-expressing patients. (*P* = 0.0305, Fig. 1c).

**Table 1:** The expression of SPEN in NPC compared with NP tissues.

Group	Cases (*n*)	SPEN expression		*χ*^2^	P^*^ value
		High expression	Low expression		
NPC	238	141 (59.2%)	97 (40.8%)	29.234	0.000
NP	54	10 (18.5%)	44 (81.5%)		

*NPC* nasopharyngeal carcinoma, *NP* nasopharyngeal epithelium.

*χ^2^ test was applied to assess the expression of SPEN in NPC and NP

**Table 2:** Correlation between the clinicopathologic characteristics and expression of SPEN in NPC.

Characteristics	Cases (*n*)	SPEN expression		*χ*^2^	P^*^ value
		High expression	Low expression		
Gender					
Male	181	108 (59.7%)	73 (40.3%)	0.056	0.812
Female	57	33 (57.9%)	24 (42.1%)		
Age (years)					
≤50	153	92 (60.1%)	61 (39.9%)	0.140	0.709
>50	85	49 (57.6%)	36 (42.4%)		
*N* classification					
N0–N1	122	47 (38.5%)	75 (61.5%)	44.502	0.000
N2–N3	116	94 (81.0%)	22 (19.0%)		
T classification					
T1–T2	110	63 (57.2%)	47 (42.8%)	5.331	0.021
T3–T4	108	78 (72.2%)	30 (27.8%)		
Clinical stage					
I–II	64	25 (39.0%)	39 (61.0%)	14.766	0.000
III–IV	174	116 (66.7%)	58 (33.3%)		

*NPC* Nasopharyngeal carcinoma, *NP* normal epithelium.

*χ^2^ test was applied to access the associations between SPEN expression and the clinicopathological parameters.

### Knockeddown expression of SPEN suppresses cell migration and invasion in vitro and inactivates PI3K/AKT and c-JUN signaling

**Fig. 2:**
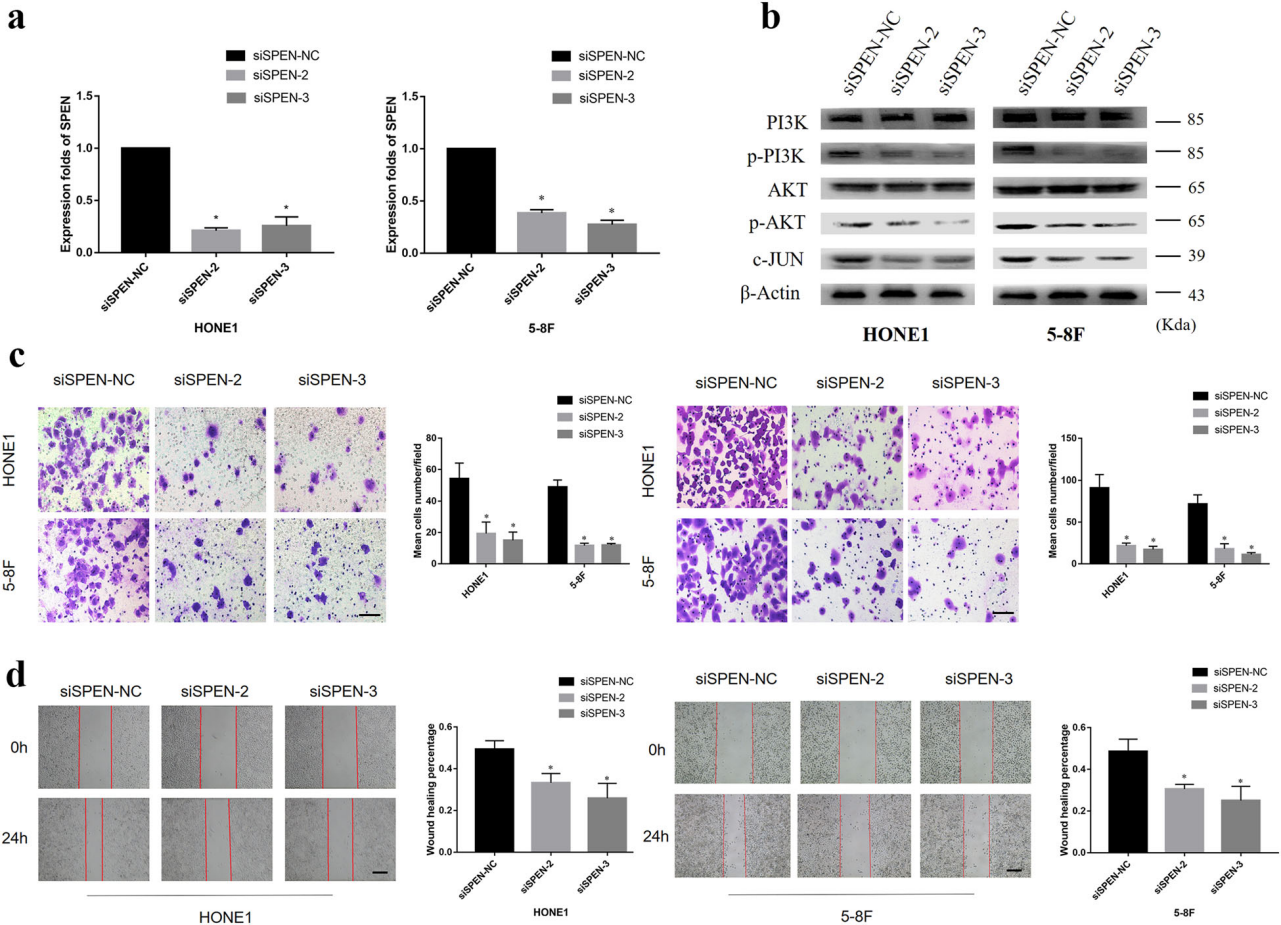
Knocked-down expression of SPEN suppresses cell migration and invasion in vitro and inactivates PI3K/AKT and c-JUN signaling. **a** qRT-PCR analysis of SPEN after SPEN silencing by siRNAs. *P < 0.05. **b** Expression levels of p-PI3K, PI3K, p-AKT, AKT, and c-JUN were determined in HONE1 and 5–8F cell lines by western blot after transfection of siRNAs for SPEN. Slience d SPEN significantly inhibited HONE1 and 5-8 F cell migration and invasion as examined by transwell and boyden assays (**c**) and wound**-**healing assay (**d**), respectively. Data are presented as mean ± SD for three independent experiments. *P < 0.05.

RNA interference was conducted to knock down *SPEN* expression in HONE1 and 5–8F cells. *SPEN* gene expression analysis by qRT-PCR confirmed that, after silencing, its expression was significantly decreased in NPC cells compared with their control cells (Fig. 2a). After *SPEN* knockdown, the expression of p-PI3K and p-AKT was largely abrogated, as well as the expression of c-JUN (Fig. 2b). In addition, Transwell, Boyden and wound-healing assays to investigate the effect of silencing *SPEN* on the migration and invasion abilities, NPC cells showed that downregulation of *SPEN* in HONE1 and 5–8F NPC cells markedly inhibited cell migration and invasion abilities (Fig. 2c, d). Taken together, these findings revealed that inhibition of SPEN decreased NPC cell migration and invasion as well as inactivated PI3K/AKT and c-JUN signaling.

### SPEN induces miR-4652-3p expression in NPC cells

**Fig. 3:**
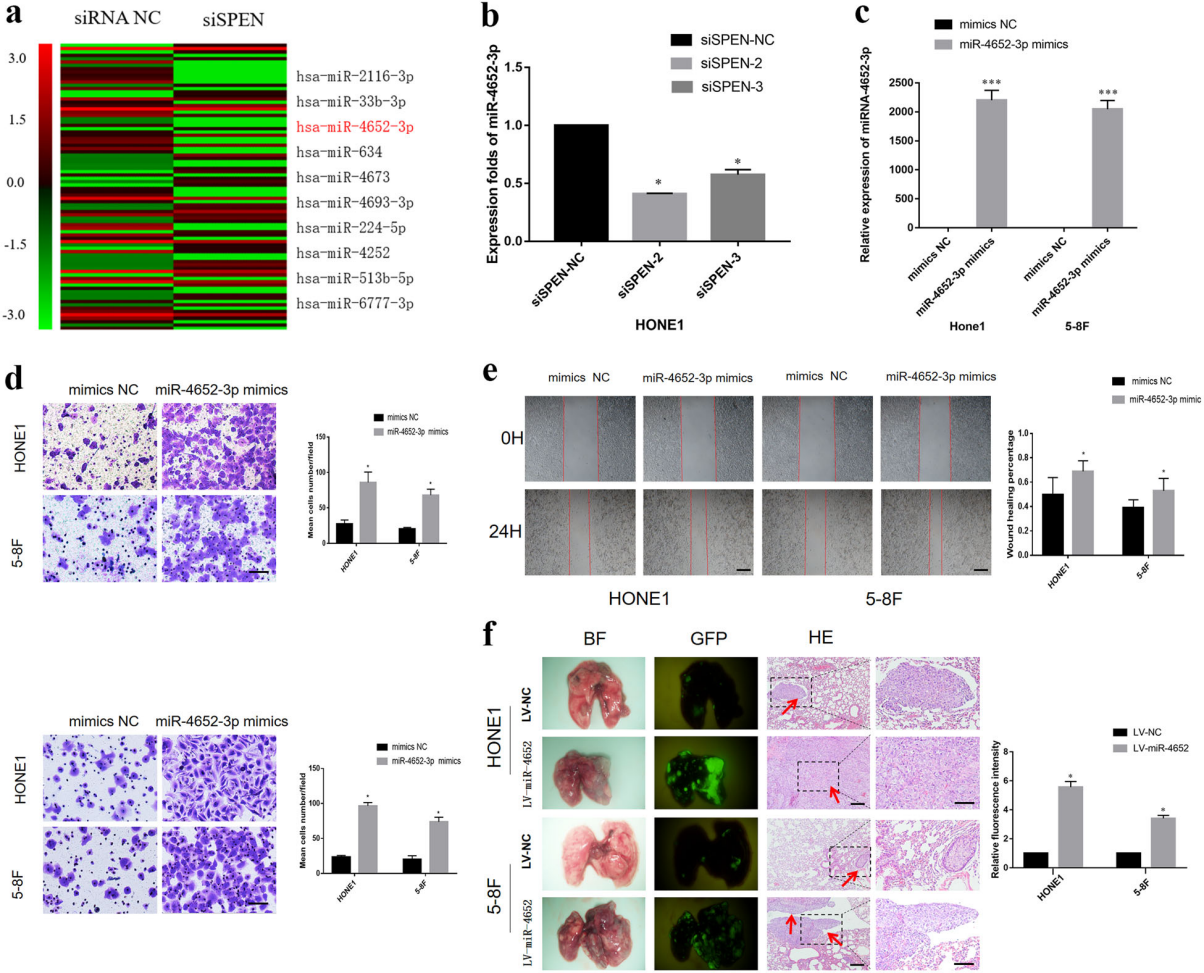
SPEN induces miR-4652-3p expression in NPC cells. **a** Expression of miR-4652-3p were downregulated after knockdown of SPEN in HONE1 cells based on a miRNA array. **b** qRT-PCR detected miR-4652-3p expression in SPEN-silenced HONE1 cells compared with HONE1-NC group. *P < 0.05. **c** miR-4652**-**3p expression was remarkably upregulated after miR-4652-3p mimics infection in HONE1 and 5-8 F. ***P < 0.001. miR-4652-3p promoted cell migration and invasion in HONE1 and 5-8F cells examined by transwell, boyden (**d**) and wound-healing assay (**e**). *P < 0.05 (**f**) Representative bioluminescence images of the lungs after tail vein injection of cells. Lung metastases were confirmed by H&E staining. Scale bars, 200 μm. Arrows indicate nests of malignant cells.

To investigate the downstream effector miRNAs regulated by SPEN, an Affymetrix 3.0 miRNA array was used to examine the differential expression of miRNAs between HONE1-si*SPEN* and HONE1-NC cells (Fig. 3a). Expression analysis by qRT-PCR confirmed that miR-4652-3p expression was downregulated by twofold or more at the mRNA level in *SPEN*-silenced HONE1 cells compared with HONE1-NC group (Fig. 3b).

### MiR-4652-3p promotes NPC cell metastasis in vitro and in vivo

HONE1 and 5–8F cells successfully transfected with miR-4652-3pmimics were used to investigate the effects of miR-465-3p on migration and invasion alterations in vitro. First, qRT-PCR analysis showed the expression level of miR-4652-3p was higher in HONE1 and 5–8F cells transfected with miR-4652–3p mimics than that in cells transfected with NC (Fig. 3c). In addition, overexpression of miR-4652-3p in HONE1 and 5–8F cells greatly promoted cell migration and invasion abilities (Fig. 3d, e). In addition, we established two NPC cell lines stably overexpressing miR-4652–3p, namely HONE1-LV-miR-4652-3p and 5-8F-LV-miR-4652-3p, by lentivirus-mediated transfection and used them for in vivo studies. Pulmonary metastasis were performed in animal models, developed by injecting NPC cell lines stably overexpressing miR-4652-3p via the tail vein of nude mice. Metastatic nodules in the lung of nude mice were detected under a fluorescence microscope and confirmed by histological analysis. More pulmonary metastases were detected in the miR-4652-3p overexpressing group than in the control (NC) group. No metastases were detected in heart, liver, spleen, kidney, brain, and other organs (Fig. 3f).

### Suppression of miR-4652-3p expression inhibits NPC cell migration and invasion

**Fig. 4:**
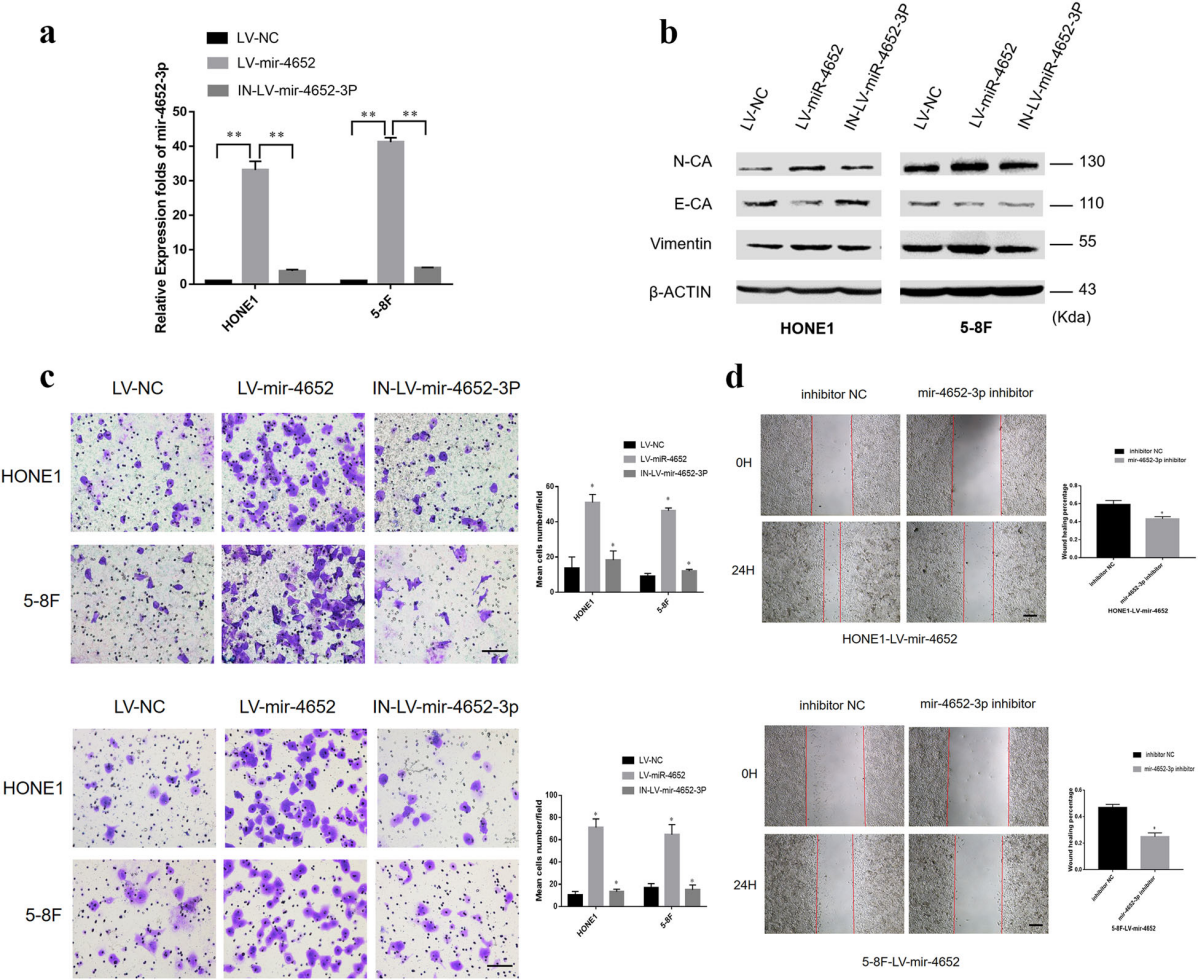
Suppression of miR-4652-3p expression inhibits NPC cell migration and invasion. **a** miR-4652-3p level in HONE1-LV- miR-4652-3p and 5-8F-LV-miR-4652-3p cell lines was largely decreased after transfection with miR-4652-3p inhibitor. ^**^P < 0.01. **b** Expression levels of E-cadherin, N-cadherin and Vimentin were determined in HONE1-LV-miR-4652-3p, 5-8F-LV-miR-4652-3p and their corresponding miR-4652-3p inhibited cell lines by western blot. β-ACTIN was used as a loading control. Transwell, Boyden assay (**c**) and wound**-**healing assay (**d**) of HONE1-LV-miR-4652-3p, 5-8F-LV-miR-4652-3p and their corresponding miR-4652-3p inhibited cell lines. Data are presented as mean ± SD for three independent experiments. ^*^P < 0.05.

The biological role of miR-4652-3p in NPC pathogenesis was further investigated by introducing miR-4652-3p inhibitors into the HONE1-LV-miR-4652-3p and 5-8F-LV-miR-4652-3p cells stably overexpressing miR-4652-3p. The expression level of miR-4652-3p was found to be elevated in the NPCLV-miR-4652-3p cells compared with LV-NC group, while miR-4652-3p inhibitors significantly reduced the miR-4652-3p expression level (Fig. 4a). Consistent with the role of miR-4652-3p in migration and invasion, inhibition of miR-4652-3p expression by its specific inhibitor suppressed cell migration and invasion in miR-4652-3p-overexpressing HONE1 and 5–8F cells (Fig. 4c, d). The analysis of the mechanism revealed that miR-4652-3p overexpression upregulated the protein expression levels of N-cadherin and vimentin, but downregulated E-cadherin protein level. On the other hand, inhibition of miR-4652-3p expression led to opposite effect on the expression levels of these epithelial–mesenchymal transition (EMT) related proteins (Fig. 4b).

### MiR-4652-3p directly targets HIPK2

**Fig. 5:**
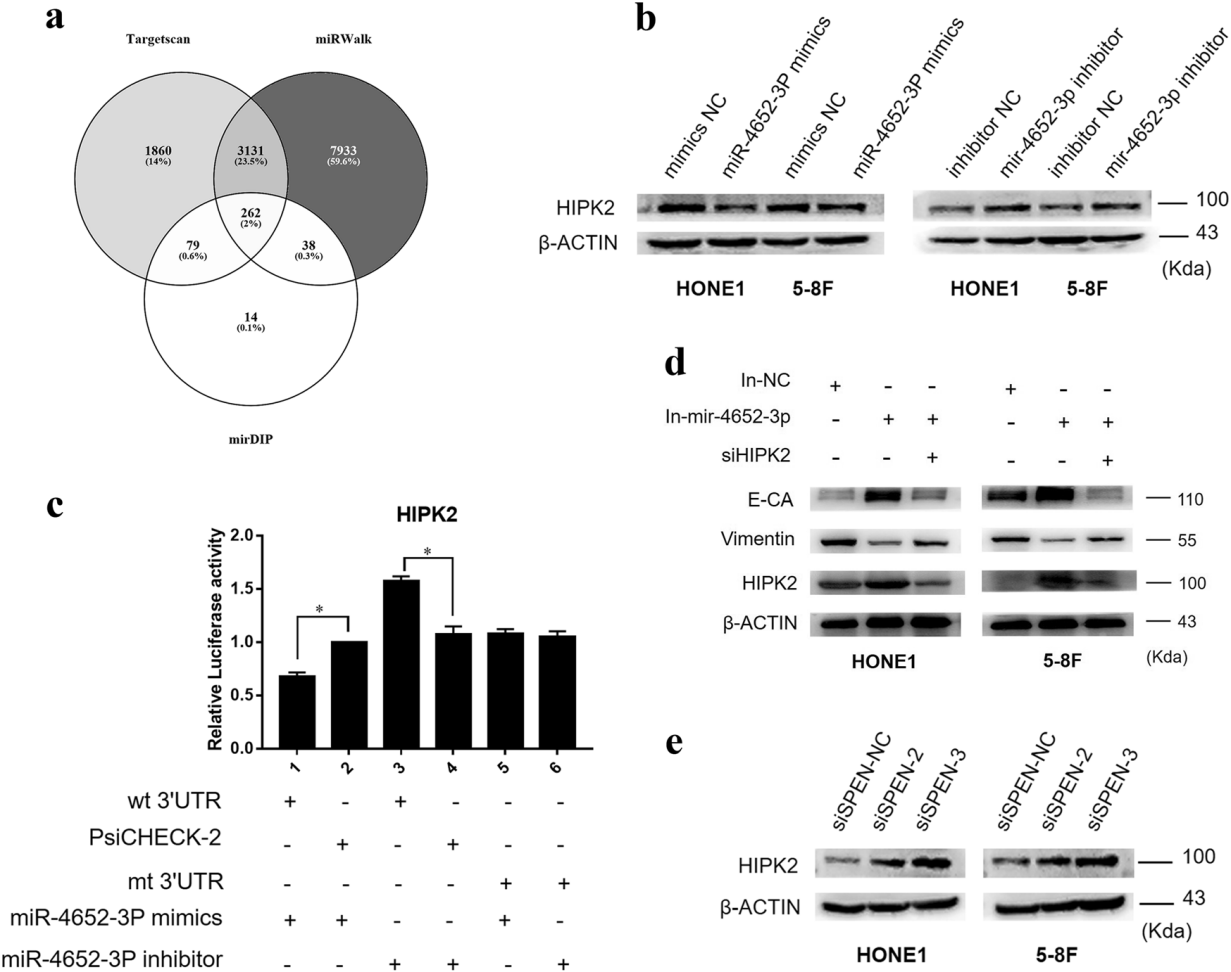
miR-4652-3p directly targets HIPK2. **a** Targetscan, miRWalk, and mirDIP were used to predict target genes of miR-4652-3p. **b** HIPK2 expression was examined in miR-4652-3p-overexpressing or miR-4652-3p-inhibited cells by western blot, β-actin was used as a loading control. **c** Luciferase reporter assay was used to determine miR-4652-3p directly targets the 3′UTR of HIPK2. ^*^P < 0.05. **d** Changes of E-cadherin, Vimentin and HIPK2 expression were detected by western blot in HONE1 and 5–8F cell lines after transfection of miR-4652-3p inhibitor or HIPK2 siRNAs. **e** Western blot of HIPK2 protein expression in HONE1 and 5–8F cells treated with SPEN siRNAs or corresponding control. β-actin served as a loading control.

To further explore the mechanisms by which miR-4652-3p promotes NPC cell migration and invasion, various miRNA target prediction software were used to predict miR-4652-3p target genes, including TargetScan, miRWalk, and mirDIP. A total of 262 potential targets were found in the above-mentioned three databases, which include HIPK2 (Fig. 5a). Western blot analysis confirmed that the HIPK2 protein level was downregulated by miR-4652-3p overexpression, and upregulated by miR-4652-3p inhibitors (Fig. 5b). Simultaneously, co-transfection of miR-4652-3pmimics and *HIPK2* 3′ untranslated region (UTR) WT sequence significantly decreased the luciferase reporter activity, whereas the miR-4652-3p inhibitor had the opposite effect. These effects on luciferase activity were abrogated when cells were co-transfected with mutated *HIPK2* 3′UTR (Fig. 5c). Furthermore, depletion of HIPK2 induced the expression of vimentin, but reduced E-cadherin expression, indicating that *HIPK2* silencing restored the EMT signaling suppression caused by inhibition of miR-4652-3p (Fig. 5d). Moreover, HIPK2 expression level was increased after the silencing SPEN in NPC cells, indicating that HIPK2 is involved in the SPEN/miR-4652-3p-induced NPC cell metastasis (Fig. 5e).

### SPEN induces miR-4652-3p expression by modulating PI3K/AKT/c-JUN signaling

**Fig. 6:**
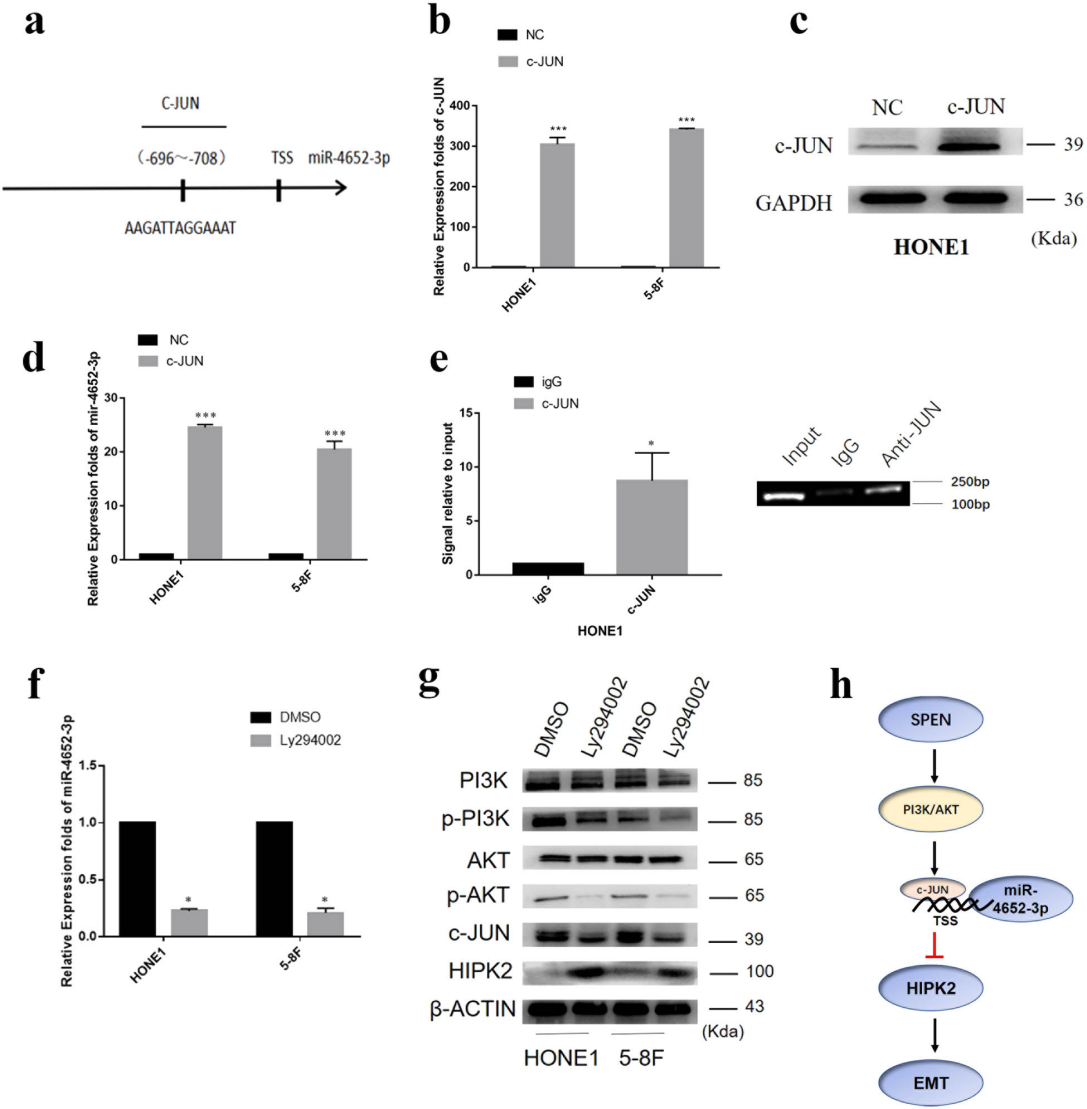
SPEN induces miR-4652-3p expression by modulating PI3K/AKT/c-JUN signaling. **a** Predicted c-JUN binding site to the promoter region of miR-4652-3p. **b** qRT-PCR of c-JUN mRNA expression in HONE1 and 5-8F cell treated with c-JUN plasmids. ^***^P < 0.001. **c** Western blot of c-JUN Protein expression in HONE1 cells transfected with c-JUN plasmids. **d** Transfection of c-JUN plasmids stimulated miR-4652-3p expression in HONE1 and 5–8F cells. ^***^P < 0.001. **e** ChIP assay to verify the binding of c-Jun to miR-4652-3p. ^*^P < 0.05. **f** mRNA expression of miR-4652-3pin HONE1 and 5-8F cell transfected with LY294002. ^*^P < 0.05**. g** Western blot detection of PI3K, p-PI3K, AKT, p-AKT, c-JUN, and HIPK2 expression in HONE1 and 5-8F cell lines treated with PI3K inhibitor LY294002. β-Actin was used as a loading control. **h** Graphic abstract depicting a proposed model for a major mechanism of SPEN in promoting NPC cell migration and invasion.

To determine the transcriptional regulatory mechanisms of miR-4652-3p expression, the University of California Santa Cruz and Profiler of Multi-Omic data online software were used to analyze a 2-kb upstream region to the transcription start site of miR-4652-3p. A potential c-JUN binding site was predicted at −696 to −708 (Fig. 6a). Accordingly, c-JUN plasmids were introduced into NPC cells, and its effect on miR-4652-3p expression was evaluated by qRT-PCR analysis and Western blot analysis (Fig. 6b, c). The results of the qRT-PCR analysis indicated that miR-4652-3p expression was greatly elevated in HONE1 and 5–8F cells transfected with c-JUN plasmids, suggesting that c-JUN acts as an upstream regulator of miR-4652-3p (Fig. 6d). The binding of c-JUN to the miR-4652-3p promoter region was further confirmed by chromatin immunoprecipitation (ChIP) analysis (Fig. 6e). In a subsequent experiment, LY294002, a specific inhibitor of PI3K, was used to block the PI3K expression in HONE1 and 5–8F cells and its effect on miR-4652-3p function was evaluated. Western blot and qRT-PCR analyses demonstrated that LY294002 decreased the levels of p-PI3K, p-AKT, c-JUN, and miR-4652-3p, but increased HIPK2 expression compared with the control groups (Fig. 6f, g). Our results showed that SPEN induced miR-4652-3p expression by activating PI3K/AKT/c-JUN signaling.

## Materials and methods

### Cell lines and cell cultures

The six NPC cell lines (5–8F, 6–10B, CNE1, CNE2, HONE1, and SUNE1) and immortalized nonmalignant human nasopharyngeal epithelial NP69 cells used in this study were obtained from the Cancer Research Institute of Southern Medical University (Guangzhou, China). The immortalized nonmalignant human nasopharyngeal epithelial cell line SXSW-1489 was purchased from SHBIO (SiXin, Shanghai, China). In this study, the NPC and SXSW-1489 cells were cultured in RPMI-1640 medium supplemented with 10% fetal bovine serum (FBS; HyClone, Logan, UT, USA). NP69 cells were cultured in a defined keratinocyte serum-free medium supplemented with epidermal growth factor (EGF, Invitrogen, Carlsbad, CA, USA).

### RNA isolation and qRT-PCR

Total RNA from each sample was quantified by the NanoDrop2000 and converted to cDNA. cDNA was added to RT 2 Profiler™ PCR Array (TAKARA, JAPAN) and ran in Real-Time PCR instrument (Applied Biosystems, Thermo Fisher Scientific, NY, USA). Primer sequence SPEN (Forward: CGAGCATTTCAAACGATATGGC; Reverse: CCATTTTGTTGACCGAGTTGTG), c-JUN (Forward: GTGCCGAAAAAGGAAGCTGG, Reverse: CTGCGTTAGCATGAGTTGGC), HIPK2 (Forward: CGGACTGGAGAAATACGCA, Reverse: ACAGATGACTGGTGCTGCTTAC), miR-4652-3p (GCGGTTCTGTTAACCCATCCCCTCA) were synthesized and purchased by Sangon Biotech (Shanghai, China).

### Western blot analysis

Whole cell lysates of NPC cells were prepared with a proteinase and phosphatase inhibitor cocktail (Roche, CA, USA). Equal amounts of proteins were resolved to 10% SDS-polyacrylamide gel electrophoresis and transferred to PVDF membranes (Millipore, Danvers, MA, USA) and blocked with 5% nonfat dry milk in Tris-buffered saline, pH 7.5. Membranes were immuno-blotted overnight at 4 °C. Protein blots were probed with primary antibodies against HIPK2 (Abcam #ab108543), E-cadherin (Cell Signaling Technology #3195), N-cadherin (Cell Signaling Technology #13116), SPEN (Abcam #ab72266), c-JUN (Abcam #ab40766), phosphor-AKT (Cell Signaling Technology #4060), AKT (Cell Signaling Technology #4691), phosphor-PI3K (Cell Signaling Technology #17366), and PI3K (Cell Signaling Technology #4292). Goat anti-rabbit IgG HRP-linked antibody (1:10,000) and goat anti-mouse IgG HRP-linkedantibody (1:10,000) were from Proteintech (Rosemont, IL, USA). Signals were visualized by chemiluminescence (Bio-rad, Hercules, California) and quantitated using a Quantity One system (Bio-Rad, Hercules, CA, USA).

### Immunohistochemical staining

Two hundred and thirty-eight (238) paraffin-embedded NPC specimens and fifty-four (54) cases of nonneoplastic nasopharyngeal mucosal tissue were collected from Nanfang Hospital of Southern Medical University without any therapy before sampling. Among 238 NPC cases, 181 cases are male and 57 are female, ranged from 20 to 84 years old of age (median 58.9 years old). The clinical process was approved by the Ethics Committees of Nanfang Hospital of Southern Medical University and the informed consent were obtained from patients. Two independent pathologists (Dr. Zhen Liu and Qingping Jiang) performed the blinded scoring. IHC staining scores were judged by the following criteria: A staining index (ranging from 0 to 12) was determined by the intensity of SPEN staining (0 = negative, 1 = weakly positive, 2 = moderate positive, 3 = strongly positive),multiplied by the proportion score of immunopositive tumor cells (0% = 0, <10% = 1, 10% to <50% = 2, 50% to <75% = 3, ≥75% = 4). A minimum of 300 epithelial cells were counted for each sample and 3 or higher scores were classified as high SPEN expression.

### RNA interference

The siRNAs specifically against SPEN and HIPK2 gene and their corresponding scrambled siRNAs were transfected into NPC cells in six-well plates using Lipofectamine 3000 transfection reagent (Invitrogen) according to the manufacturer’s instructions. The scramble small-interfering RNA (NC) and the siRNAs targeting SPEN(#2, 5′-CCAAGATCGTACATATTAT-3′; #3, 5′-GGATCATGGTGCATCCACA-3′), and HIPK2 (#1, 5′-GCUCACGGAAGCCAUUAUATT-3′, #2, 5′-GCGGACCACACAACCUAAUTT-3′) were synthesized and purchased from Ribobio (Guangzhou, China).

### In vitro cell migration, invasion and wound-healing assays

For migration assay, 1 × 10^4^ cells in serum-free culture medium were added to the upper chamber, and the lower chamber was filled with 10% FBS culture media. Micro pore size of transwell membrane is 8 μm. After incubation, the filter was fixed with methanol, stained with crystal violet solution, and counted under a microscope in three random fields (×200).

For invasion assay, transwell membranes were pre-coated with 35 μl diluted matrix matrigel (BD Biosciences, USA) for 30 min. 1 × 10^5^ Cells adhering to the lower surface were counted the same way with the cell migration assay. Cells that migrated and invaded were quantified by counting cells in three random fields per filter.

For wound-healing assay, 4 × 10^6^ cells were seed to confluence in a six-well plate, then cultured with serum-free medium. Artificial wound tracks were created by straight scraping confluent cell with a pipette tip. The ability of the cells to migrate into the wound area was assessed at 0 and 24 h after scratching. *miRNA array following* siSPEN Interference of SPEN in HONE1 cells was sent to Shanghai OE Biotech. Co., Ltd. (Shanghai, China) for miRNA biochip correlation analysis. The biochip used in this study was the SurePrint Agilent Human miRNA Microarrays (Release 21.0, ID:070156) obtained from (Agilent Technologies, Santa Clara, CA, USA).

### Establishment of NPC cell lines stably overexpressing miR-4652-3p

HONE1 and 5–8F cells were infected with a lentiviral expression vector carrying miR-4652-3p, which was constructed by GeneChem Co., Ltd. (Shanghai, China). MiR-4652-3p overexpressing cells with green fluorescent protein signals were selected for further experiments by screening with puromycin. Total RNA was extracted from these cells and reverse transcribed into cDNA to measure the infection efficiency by qRT-PCR analysis.

### In vivo metastasis assay in nude mice

Five female BALB/c nude mice (4-week old) were randomly divided into four groups and injected with HONE1/5-8F-miR-4652-3P or HONE1/5-8F-miR-control cells. Briefly, 1 × 10^6^ cells were injected intravenously through the tail vein of each nude mouse in a laminar flow cabinet. Four weeks after injection, the mice were sacrificed and examined for routine tissue processing. Lung tissues were then fluorescently imaged using the LT-9MACIMSYSPLUS whole-body imaging system (Light Tools Research, Encinitas, CA, USA). Subsequently, the Image J software (NIH, Bethesda, MD, USA) was used to quantifythe fluorescence signal intensity emitted from lung tissues. All animal experiments were conducted according to the standard institutional guidelines of Guangzhou Medical University. All animal experimental procedures were performed according to the Guidelines for the Care and Use of Laboratory Animals (NIH publications Nos. 80–23, revised 1996).

### Dual luciferase reporter assay

The prediction software used predicted that *HIPK2* is probably a direct target of miR-4652-3p. The 3′UTR fragment of *HIPK2* was amplified using PCR primers and cloned into the psiCHECK-2 vector. Site-directed mutagenesis of the miR-4652-3p binding site at the *HIPK2* 3′UTR was performed using the GeneTailor system (Invitrogen). The wild (wt) or mutant (mt) type of the *HIPK2* 3′UTR vector, psiCHECK-2 were co-transfected into the cells with miR-4652-3p mimics or inhibitors. The tests were independently performed in triplicate using a dual luciferase assay kit (Promega Corp., Madison, WI, USA) according tothe manufacturer’s instructions.

### Chromatin immunoprecipitation (ChIP) assay

ChIP analysis was performed using a ChIP assay kit (catalog: 17-371; Millipore, Billerica, MA, USA) according to the manufacturer’s instructions to determine whether c-JUN binds to the promoter of miR-4652-3p. First, cells were transfected with a c-JUN plasmid and fixed with 1% formaldehyde. The cross-linked DNA is sonicated, cut to a length of 200–1000 base pairs, and then subjected to an immunoselection process, which requires the use of an anti-c-JUN antibody (1:50; Cell Signaling Technology (CST), Inc., Danvers, MA, USA). Ultimately, the agarose gel electrophoresis results reveal whether the DNA fragment of the putative c-JUN binding site was present in the miR-4652-3p promoter.

### Statistical analysis

Statistical analyses were performed using the SPSS 16.0 software (IBM Corp., Armonk, NY, USA) and GraphPad Prism v5.0 software (GraphPad Software Inc., La Jolla, CA, USA). Data are presented as the mean ± SEM. For migration and invasion assays, statistical significance was determined using the Student’s two-tailed *t* test for two groups and one-way analysis of variance (ANOVA) for multiple groups. Immunohistochemistry analysis results were analyzed by the chi-square (*χ*^2^) test. Survival analysis was performed using the Kaplan–Meier method. A *P* value of < 0.05 was considered as statistically significant. *P < 0.05, **P < 0.01, and ***P < 0.001.

## Discussion

SPEN has been reported to play a dual role in tumor development^8–10^. However, its biological function and molecular mechanism in NPC have not been elucidated. In this study, we first observed the upregulated mRNA expression of SPEN in NPC cells compared with immortalized nasopharyngeal epithelial (NP) cell lines (NP69 and SXSW-1489). In addition, IHC analysis revealed elevated SPEN protein expression level in NPC tissues compared with NP tissues. High expression of SPEN was identified as an independent predictor and cause of the poor outcome of NPC patients. Further, knocking down SPEN suppressed the migration and invasion of NPC cells. These findings was similar to Légaré and Liu et al.’s reports in breast cancer and ovarian cancer^9,10^, which demonstrated that dysregulation of SPEN was significantly involved in the development of NPC, suggesting that it plays a potential tumor metastasis promoter in NPC.

Molecular mechanisms underlying the migration and invasion of tumor cells have been intensively studied^17,18^. PI3K/AKT is a key oncogenic signal that had been widely documented to activate EMT pathway and thus promote tumor migration, invasion, and metastasis^19,20^. In previous investigation, we also observed that dysregulated PI3K/AKT and its downstream EMT participated in some tumor-related gene-mediated NPC migration, invasion, and metastasis^6,12^. In this study, analog to Liu et al.’s data^10^, we found that suppressing SPEN reduced PI3K/AKT and its downstream EMT signal. Furthermore, c-Jun, an oncogenic transcription factor was also decreased in SPEN-suppressed NPC cells. These data further supported SPEN as a tumor metastasis promoter in NPC.

MiRNAs as intermediate regulators have been widely shown to participate in tumor-related gene-mediated signal network^21–23^. In previous studies, we also observed that miR-133a-3p, miR-5188, miR-3188, miR-374a, and miR-296-3p, respectively were modulated by VPS33B, HBx, FOXO1, PDCD4, and HDGF involving in the tumor pathogenesis induced by these genes^5,6,12,13,20,24,25^. In order to further explore the molecular mechanism of metastasis induced by SPEN in NPC, Affymetrix miRNA array and qRT-PCR were used to identify the differential miRNAs in SPEN-knocking down cells. The data revealed that miR-4652-3p was a significantly positive-regulator of SPEN and might function as a tumor-promoted role in NPC cells. Consistent with our speculation, miR-4652-3p was found as a tumor metastasis promoter accelerating the metastasis of NPC. However, this data were not consistent with Li’*s report for* miR-4652-3p in malignant meningioma^26^, which suggests the complexity of miR-4652-3p in tumors.

In order to explore the mechanisms by which miR-4652-3p enhanced NPC cell migration, invasion and metastasis, bioinformatics were used to predict target genes through Targetscan, miRWalk, and mirDIP. Two hundred and sixty-two potential targets were found in all the above three databases, including HIPK2. The serine/threonine kinase HIPK2, which was identified as a potential tumor suppressor in human neoplasms, is involved in transcriptional regulation and apoptosis^27–32^. Furthermore, HIPK2 inhibition was reported to promote EMT and subsequent cell invasion in bladder cancer^27^. In this study, we found that miR-4652-3p directly targeted HIPK2 and HIPK2 silencing abrogated the EMT signaling alteration caused by miR-4652-3p interference. These data demonstrated that miR-4652-3p targets HIPK2 to activate EMT signal and function as a promoter of tumor metastasis.

In prior study, we have shown that SPEN induces mir-465-3p expression, However, the mechanism by which SPEN regulates miR-4652-3p expression has not been elucidated. It is well known that transcript factor-mediated miRNA transcription expression has been widely documented in tumors^11,20,25^. In this study, we first predicted the possible transcription factors binding to the miR-4652-3p promoter. Notably, among them, c-JUN was predicted as a potential transcription factor of miR-4652-3p. Overexpressed c-JUN markedly increased the expression of miR-4652-3p. In addition, the ChIP analysis indicated that c-JUN binds to the miR-4652-3p promoter.

C-JUN is an oncogenic transcription factor known as a key regulator of major biological processes^33^ and recognized as a downstream positive regulator of PI3K/AKT signaling^15,34–36^. In previous studies, we also observed that c-JUN was induced by PI3K/AKT signal to modulate the expression of miRNAs expression in tumors including NPC^14,24,37^. Interestingly, we had observed that SPEN positively regulated the expression of PI3K/AKT/c-JUN and thus speculated that SPEN modulates the miR-4652-3p/HIPK2-mediated EMT signaling through the PI3K/AKT/c-JUN pathway. In line with this notion, suppressing p-PI3K with its specific inhibitor LY294002 reduced the activation of p-AKT/c-JUN, which further downregulated miR-4652-3p expression, thereby increasing the HIPK2-induced inhibition of EMT signaling. Finally, we confirmed that SPEN was negatively correlated with HIPK2 protein expression in NPC cells and immortalized nasopharyngeal NP69 cells.

Overall, our study demonstrated for the first time that elevated SPEN might be used as a useful prognostic biomarker in NPC. Specifically, it activates PI3K/AKT/c-JUN to modulate miR-4652-3P/HIPK2 axis, which in turn activates EMT signaling and promotes NPC metastasis. Our findings reveal a novel pathway involved in NPC pathogenesis and broaden our understanding of NPC metastasis, which offer treatment options for improving the survival of patients with NPC.

## Data Availability

The data generated, used, and analyzed in the current study are available from the corresponding author in response to reasonable request.
